# House Dust Mite and Cat Dander Extract Induce Asthma-Like Histopathology with an Increase of Mucosal Mast Cells in a Guinea Pig Model

**DOI:** 10.1155/2023/9393497

**Published:** 2023-01-31

**Authors:** Patricia Ramos-Ramírez, Jielu Liu, Sofia Mogren, Joshua Gregory, Malin Noreby, Anne Petrén, Ying Lei, Henric Olsson, Marianne van Hage, Jukka Kervinen, Lars Hellman, Cecilia Andersson, Gunnar Nilsson, Mikael Adner

**Affiliations:** ^1^Experimental Asthma and Allergy Research Unit, Institute of Environmental Medicine (IMM), Karolinska Institutet, Stockholm, Sweden; ^2^Respiratory Cell Biology, Lund University, Lund, Sweden; ^3^Department of Medicine Solna, Division of Immunology and Allergy, Karolinska Institutet and Karolinska University Hospital, Stockholm, Sweden; ^4^Translational Science and Experimental Medicine, Research and Early Development, Respiratory & Immunology, BioPharmaceuticals R&D, AstraZeneca, Gothenburg, Sweden; ^5^Tosoh Bioscience LLC, King of Prussia, PA, USA; ^6^Department of Cell and Molecular Biology, Uppsala University, Sweden

## Abstract

**Background:**

Asthma is a chronic inflammatory disease with structural changes in the lungs defined as airway remodelling. Mast cell responses are important in asthma as they, upon activation, release mediators inducing bronchoconstriction, inflammatory cell recruitment, and often remodelling of the airways. As guinea pigs exhibit anatomical, physiological, and pharmacological features resembling human airways, including mast cell distribution and mediator release, we evaluated the effect of extracts from two common allergens, house dust mite (HDM) and cat dander (CDE), on histopathological changes and the composition of tryptase- and chymase-positive mast cells in the guinea pig lungs.

**Methods:**

Guinea pigs were exposed intranasally to HDM or CDE for 4, 8, and 12 weeks, and airway histology was examined at each time point. Hematoxylin and eosin, Picro-Sirius Red, and Periodic Acid-Schiff staining were performed to evaluate airway inflammation, collagen deposition, and mucus-producing cells. In addition, Astra blue and immunostaining against tryptase and chymase were used to visualize mast cells.

**Results:**

Repetitive administration of HDM or CDE led to the accumulation of inflammatory cells into the proximal and distal airways as well as increased airway smooth muscle mass. HDM exposure caused subepithelial collagen deposition and mucus cell hyperplasia at all three time points, whereas CDE exposure only caused these effects at 8 and 12 weeks. Both HDM and CDE induced a substantial increase in mast cells after 8 and 12 weeks of challenges. This increase was primarily due to mast cells expressing tryptase, but not chymase, thus indicating mucosal mast cells.

**Conclusions:**

We here show that exposure to HDM and CDE elicits asthma-like histopathology in guinea pigs with infiltration of inflammatory cells, airway remodelling, and accumulation of primarily mucosal mast cells. The results together encourage the use of HDM and CDE allergens for the stimulation of a clinically relevant asthma model in guinea pigs.

## 1. Introduction

Asthma is a chronic inflammatory disease with airway hyperresponsiveness and structural changes in the lungs defined as airway remodelling [1]. Mast cells are central effector cells in the pathobiology of both allergic T2-driven and non-T2-mediated asthma, but how these cells mediate inflammatory cell infiltration and remodelling is much less known. Upon activation, these cells release mediators that induce bronchoconstriction and cause inflammatory cell recruitment and activation which may cause structural changes in the airways. As the mouse accumulates very few mast cells in distal airways and lung parenchyma [2], we recently developed an asthma model in guinea pigs [3] which shows similar anatomy, physiology, and pharmacology to that of human disease [4, 5]. This granted mast cell interactions with important inflammatory and immunological reactions that are absent in mouse models of asthma. With a 7-week protocol, we have shown antigen-induced bronchoconstriction, airway hyperresponsiveness, airway inflammation, and remodelling, together with a marked increase of mast cells [3]. However, how these changes developed over time remained to be elucidated.

Due to strong mast cell responses, guinea pigs have been attractive for modelling airway diseases and particularly for antigen-induced contractions [5, 6]. Mast cells are typically divided into two phenotypes, mucosal mast cells that, in the human, contain tryptase but no chymase in their granules (MC_T_) and connective tissue mast cells that contain both tryptase and chymase in their granules (MC_TC_) [7]. In marked contrast, in most rodents, such as a mouse and rat, mucosal mast cells only express chymases and no tryptases [8, 9] whereas the connective tissue mast cells express both tryptases and chymases [8].

The presence of mast cell phenotypes and if their ratio or composition changes over time after allergen challenges have not yet been investigated in the guinea pig lungs. As we, in our previous study, showed the increase of mast cells in the guinea pig asthma model [3], it is of specific interest to determine the mast cell phenotypes of this increase.

To investigate the airway inflammation, remodelling, and mast cell features over time, we exposed guinea pigs to repeated challenges using clinically relevant airborne allergens over 4, 8, and 12 weeks. Because patients with allergic asthma symptoms are frequently sensitized to at least one indoor allergen, such as house dust mites or cat dander [10, 11], we chose to test these allergens in the present study. Structural changes in the airway walls, such as an increase of the smooth muscle layer, goblet cell hyperplasia, and subepithelial collagen deposition, were investigated together with the inflammatory cell infiltrate and the number of mast cells using conventional histological techniques. Moreover, to distinguish mast cell phenotypes, we used specific antibodies against two common mast cell-specific granule proteases, tryptase and chymase.

## 2. Materials and Methods

### 2.1. Animals

Male Dunkin-Hartley guinea pigs (400-450 g) were obtained from Envigo (Netherlands) and maintained on a 12/12-hour light/dark cycle with food and water *ad libitum*. Animal experiments were carried out in strict accordance with both Swedish and EU legislations on animal welfare. All the experiments were approved by the Stockholm ethics committee for animal research (N143/14).

### 2.2. Sensitization and Challenge Protocols

**Figure 1:**
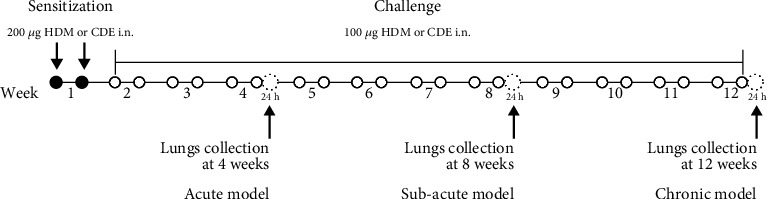
Sensitization and challenge protocols. Experimental models of acute (4 weeks), subacute (8 weeks), and chronic (12 weeks) HDM- or CDE-induced allergic airway inflammation in guinea pigs. The protocol for allergen exposure involved a sensitization phase (black dots) followed by a challenge phase (open dots).

Guinea pigs were sensitized twice through intranasal instillation of 200 *μ*g of either HDM (Greer, Lenoir, NC, USA) or CDE (Greer, Lenoir, NC, USA), prepared as described previously [12], in 300 *μ*L of PBS (Figure 1). On the challenge days, the guinea pigs were exposed to 100 *μ*g of either HDM or CDE twice per week for 4, 8, or 12 weeks (Figure 1) under light anaesthesia with 5% isoflurane. Intraperitoneal treatment with salbutamol (3 mg/kg) was applied prior to each exposure beginning with the first challenge dose to avoid possible anaphylactic reaction. Control animals were given intranasal PBS over the same time of the study.

### 2.3. Collection of Lung Tissue

One day after the completion of allergic induction protocols (4, 8, or 12 weeks), guinea pigs were euthanized with an overdose of pentobarbital. The left lung was inflated with 10% neutral-buffered formalin (Sigma-Aldrich, Germany) to a pressure of 28-30 cmH_2_O, excised and transferred to formalin for 24 hours. The right lung was inflated with Carnoy's fixative (60% ethanol, 30% chloroform, and 10% acetic acid) and maintained for 5 hours at room temperature. After fixation, both lungs were transferred to 70% ethanol and stored at +4°C for later histological processing.

### 2.4. Histological Staining and Morphometric Analysis

Caudal lobes from either the left or the right lung were dehydrated and embedded in paraffin for sagittal sectioning. Sections from the caudal left lobe were stained with hematoxylin and eosin (H&E) (Histolab, Askim, Sweden) to visualize inflammatory cells; Picro-Sirius Red staining (Abcam, Cambridge, UK) was performed to evaluate the collagen deposition and Periodic Acid-Schiff (PAS; Sigma-Aldrich) to examine the mucus-producing cells. To visualize mast cells, caudal right lung sections were stained with Astra blue (Sigma-Aldrich). Histopathological analysis was achieved blinded to the experimental groups. Stained airways were randomly selected under low-power light microscopy (40x magnification), and photomicrographs were captured with an Olympus UC50 camera (Olympus Australia, Melbourne, VIC, Australia) attached to an Olympus BX51 microscope. Surface areas (*μ*m^2^) of inflammatory infiltrate, airway smooth muscle layer, and subepithelial collagen were adjusted by the length of the corresponding basement membrane (BM) perimeter (*μ*m). PAS-positive cells were enumerated within the airway epithelial cells layer, whereas Astra blue-stained mast cells were counted in the subepithelial region. Goblet cells and mast cells were quantified under a microscope and expressed as the number of cells per mm BM. All measurements were conducted using ImageJ software (version 1.51j8, Wayne Rasband, National Institutes of Health, USA).

### 2.5. Tryptase and Chymase Immunostaining of Mast Cells

The recombinant guinea pig chymase used to raise specific antibodies was produced using the coding region retrieved from the Swiss Prot/Tr EMBL database (P85201). The guinea pig coding region was expressed in baculovirus-infected insect cells and purified as described in [13]. Polyclonal antibodies for guinea pig chymase were raised in rats by initial immunisation and two booster injections over six weeks (Innovagen AB, Lund, Sweden). Left caudal lobes were fixed in PFA and embedded in paraffin. Sections of 5 *μ*m thickness were used for immunohistochemical staining for tryptase (1 : 100, clone: G3, Cat no: MAB1222; Chemicon, Temecula, CA), and consecutive sections were stained for chymase (1 : 700). Sections were processed with antigen retrieval (EnVision FLEX, Target Retrieval Solution, low pH ref: K8005 for tryptase and high pH ref: K8004 for chymase; Dako, Glostrup, Denmark) in a PT link prior to staining. Binding of primary antibodies (Lot 1328707A, Innovagen) was visualized using the Dako REAL Envision detection system (Peroxidase/DAB+, Rabbit/Mouse, K5007; Dako) according to the manufacturer's directions. Sections were counterstained with hematoxylin and mounted in Pertex (Histolab). Positive staining was quantified manually on blinded sections and expressed as the number of cells/area of tissue. The tissue area was quantified using computerised image analysis using ImageScope (ScanScope CS, Aperio) and an automatic slide scanner (Olympus). Three randomly chosen airways and five randomly chosen fields of view of parenchyma (20x magnification) were quantified per animal. Negative controls (omitting the primary antibody) and isotype controls (Dako) were used to evaluate antibody specificity.

### 2.6. Statistical Analysis

All data were analysed by using GraphPad Prism software (version 9.4.1(681), GraphPad Inc, La Jolla, CA, USA) and are presented as the mean ± SD. The normality of the data was checked using the D'Agostino-Pearson test. Significant differences among the groups were detected by one-way ANOVA followed by Tukey's post hoc test for multiple comparisons. Statistical significance was defined as *P* < 0.05.

## 3. Results

### 3.1. Inflammation and Increase of Smooth Muscle Layer/Mass

**Figure 2:**
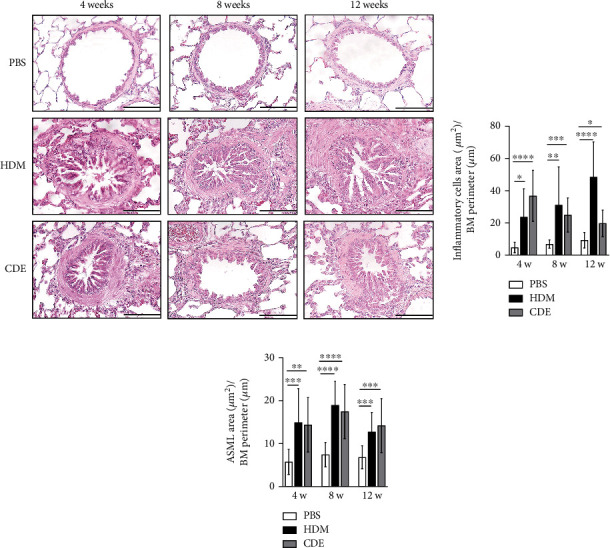
Guinea pigs exhibit a marked inflammatory reaction and remodelling to clinically relevant allergens, HDM and CDE. (a) Representative histological sections of H&E-stained lungs. (b) Analysis of infiltration of inflammatory cells infiltrated into the airway subepithelial region and (c) airway smooth muscle thickness. All measured areas (*μ*m^2^) were adjusted by the basement membrane (BM) perimeter (*μ*m). Data are the mean ± SD of *n* = 4-5 animals per group. Scale bars correspond to 100 *μ*m. ^∗^*P* < 0.05, ^∗∗^*P* < 0.01, ^∗∗∗^*P* < 0.001, and ^∗∗∗∗^*P* < 0.0001. ASML: airway smooth muscle layer; BM: basement membrane; HDM: house dust mite extract; CDE: cat dander extract.

To induce asthma-like symptoms, guinea pigs were treated intranasally twice per week for 4, 8, or 12 weeks with HDM or CDE allergens, and histological analyses were conducted on lung tissues collected 24 hrs after the last exposure (Figure 1). Lung tissue sections stained with H&E revealed that HDM and CDE induced a marked infiltration of inflammatory cells around the proximal and distal airways and the vasculature, as compared to PBS-treated controls at all three time points (Figures 2(a) and 2(b)). Examining the structural features of the airways showed that both HDM and CDE caused a marked thickening of the airway smooth muscle layer as compared to PBS controls at all three time points (Figures 2(a) and 2(c)).

### 3.2. Increase of Subepithelial Fibrosis and Goblet Cells

**Figure 3:**
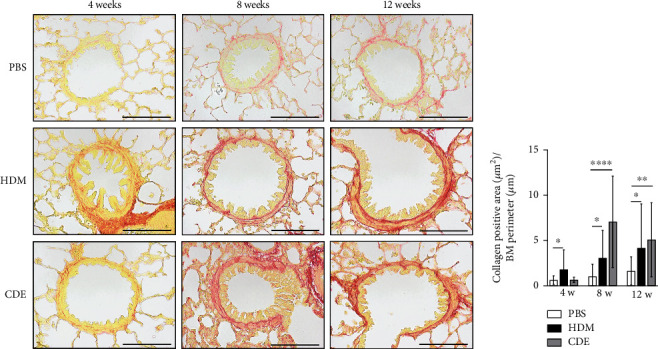
Guinea pigs exposed to HDM and CDE elicit hallmark features for airway remodelling. (a) Representative histological sections of Picro-Sirius Red-stained lungs and (b) analysis of collagen accumulation within the airway subepithelial region. Collagen-positive areas (*μ*m^2^) were adjusted by the BM perimeter (*μ*m). Data are mean ± SD of *n* = 4-5 animals per group. Scale bars correspond to 100 *μ*m. ^∗^*P* < 0.05, ^∗∗^*P* < 0.01, and ^∗∗∗∗^*P* < 0.0001. BM: basement membrane; HDM: house dust mite extract; CDE: cat dander extract.

**Figure 4:**
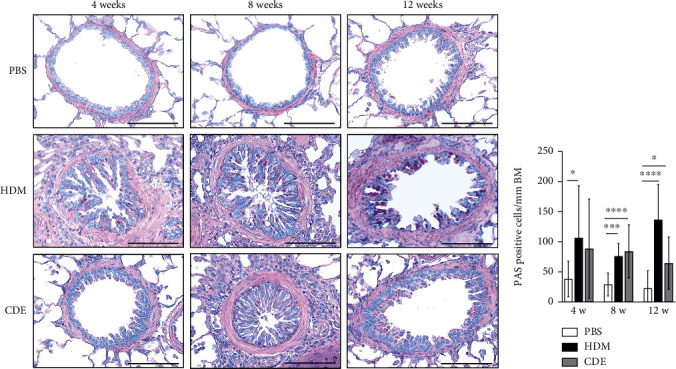
Guinea pigs exposed to HDM and CDE exhibit goblet cell hyperplasia in the airways. (a) Representative histological sections of PAS-stained lungs and (b) quantification of goblet cells expressed as cell number/BM perimeter (mm). Data are mean ± SD of *n* = 4-5 animals per group. Scale bars correspond to 100 *μ*m. ^∗^*P* < 0.05, ^∗∗∗^*P* < 0.001, and ^∗∗∗∗^*P* < 0.0001. BM: basement membrane; HDM: house dust mite extract; CDE: cat dander extract.

To investigate the effect of HDM and CDE on subepithelial collagen deposition in guinea pigs, lung tissues were stained with Picro-Sirius Red. Morphometric analysis revealed that HDM allergen induced an augmented subepithelial collagen accumulation as compared to the age-matched PBS controls at all three time points (Figure 3). Although exposure to CDE had no effect on subepithelial fibrosis after 4-week exposure, a marked collagen deposition was found in the airways of guinea pigs exposed to CDE for eight weeks that was maintained after 12 weeks (Figure 3). Assessment of the effects of exposure to HDM on the number of mucus-producing cells demonstrated a significantly increased number of PAS-positive goblet cells in the airways at all three time points. Following CDE exposure, the increase in goblet cells was significant after 8 and 12 weeks (Figure 4).

### 3.3. Increase of Tryptase- and Chymase-Expressing Mast Cells

**Figure 5:**
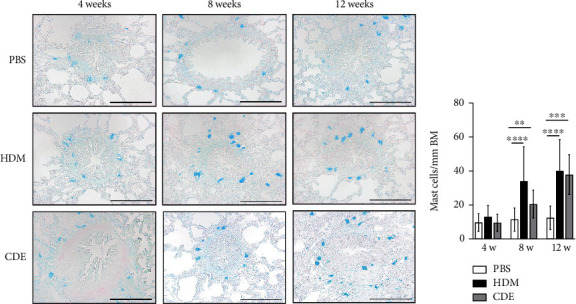
Repetitive allergen exposure induces a marked accumulation of mast cells in guinea pig airways. (a) Representative histological sections of Astra blue-stained lungs, and (b) quantification of mast cells within the airway subepithelial region. Mast cells were expressed as cell number/BM perimeter (mm). Data are mean ± SD of *n* = 4-5 animals per group. Scale bars correspond to 100 *μ*m. ^∗∗^*P* < 0.01, ^∗∗∗^*P* < 0.001, and ^∗∗∗∗^*P* < 0.0001. BM: basement membrane; HDM: house dust mite extract; CDE: cat dander extract.

To visualize airway mast cells, lung sections were treated with Astra blue staining, which is a cationic dye binding to heparin stored in mast cell granules [14, 15]. After four weeks of allergen exposure, neither HDM nor CDE elicited a significant change in the number of mast cells as compared to PBS-treated controls. In contrast, both allergens induced a marked increase of mast cells upon 8- and 12-week exposure (Figure 5).

**Figure 6:**
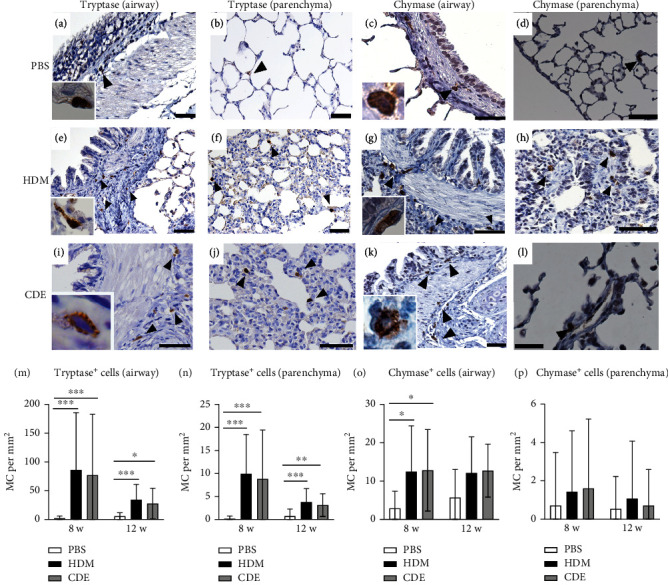
Repetitive allergen exposure induces a marked accumulation of mast cells in guinea pig airways and parenchyma as detected with immunohistochemical staining against tryptase and chymase. Micrographs of immunohistochemical staining of tryptase (a, b, e, f, i, and j) and chymase (c, d, g, h, k, and l) in airways and parenchyma, 8 weeks after challenge. PBS-treated animals (a–d); HDM (e–h); CDE (i–l). Quantification of tryptase-positive MCs in bronchi (m) and alveolar parenchyma (n) and chymase-positive MCs in bronchi (o) and alveolar parenchyma (p) expressed as MC per mm^2^. Three airways and five fields of view of parenchyma (20x magnification) were quantified per animal. Data are mean ± SD of *n* = 4-5 animals per group. ^∗^*P* < 0.05, ^∗∗^*P* < 0.01, and ^∗∗∗^*P* < 0.001. Scale bar: 50 *μ*m. High magnification micrographs (60x) are shown as inserts in (a, c, e, g, i, and k). HDM: house dust mite extract; CDE: cat dander extract.

To further define and quantify how the increase of mast cells after 8- and 12-week exposure related to different mast cell phenotypes, i.e., tryptase- or chymase-positive cells, lung sections were immunohistochemically stained on consecutive sections detecting tryptase or chymase [16] (Figure 6). At the basal level, both tryptase- and chymase-positive mast cells were found in the airways and parenchyma. In the airways that were exposed to PBS for 8 weeks or 12 weeks, the numbers of tryptase^+^ mast cells were 2.2 ± 4.0/mm^2^ and 5.5 ± 6.0/mm^2^, respectively, and of chymase^+^ mast cells, 2.9 ± 4.5/mm^2^ and 5.7 ± 7.4/mm^2^, respectively, with no significant difference between tryptase- and chymase-positive mast cells. However, as compared to mast cells in the airways, significantly less mast cells were observed in the parenchyma for both tryptase^+^ (0.2 ± 0.6/mm^2^ and 0.7 ± 1.6/mm^2^ in 8- and 12-week control animals) and chymase^+^ cells (0.7 ± 2.8/mm^2^ and 0.5 ± 1.7/mm^2^ in 8- and 12-week control animals). There were neither significant differences between tryptase- and chymase-positive cells in the airways nor in the parenchyma. In addition, most chymase^+^ cells were found to be also tryptase^+^ in consecutive sections, suggesting that the mast cells identified were mainly double-positive for tryptase and chymase.

Both HDM and CDE induced a marked expansion of tryptase^+^ mast cells in airways and alveolar parenchyma after 8-week (40- to 60-fold increase) and 12-week (4- to 6-fold increase) exposure (Figure 6). The amount of chymase^+^ mast cells was increased to a much less degree after 8 weeks (4-fold). No significant increase of chymase^+^ cells was found in the airways after 12-week allergen exposures and in the parenchyma. Thus, allergen challenges primarily induced an increase in tryptase-only positive mast cells, resembling mucosal mast cells.

## 4. Discussion

In the present study, we show that repeated airway exposure to HDM and CDE in guinea pigs developed asthma-like histopathology in the lungs. Both HDM and CDE exposure triggered peribronchovascular infiltration of inflammatory cells and increased airway smooth muscle thickness, subepithelial collagen deposition, and goblet cell hyperplasia. Most of these changes appeared during the first 4 weeks after allergen exposure and were present during the 8- and 12-week further period of the study. The number of mast cells, which was not altered after 4 weeks, was markedly increased after 8 and 12 weeks. The increase in mast cells, both around the airways and in the parenchyma, was dominated by cells that expressed tryptase but not chymase. The findings together provide evidence that HDM or CDE induced airway inflammation and remodelling, with an increase of mast cells in the guinea pig lung, resembling the pathology typical for asthma.

Both HDM and CDE induced a marked airway inflammation after 4, 8, and 12 weeks of exposure, which is in accordance with our previously published guinea pig asthma model using HDM as an antigen applied intranasally during a 7-week period [3]. Furthermore, in an OVA-driven asthma model, infiltration of cells in the airways was detected at early time points [6], and infiltrated cells were also reported in the airways up to 8 and 12 weeks after OVA exposure [17, 18]. This infiltration of inflammatory cells around proximal and distal airways after exposure to allergens is a typical feature observed in asthma [19, 20].

Both HDM and CDE allergens caused a marked thickening of the airway smooth muscle in large and small airways during the first 4 weeks that was maintained throughout the study. An increase in thickness of the airway smooth muscle has also been shown earlier in a guinea pig asthma model after a 5-week protocol with aerosol exposure every third day with increasing doses of OVA [21]. In contrast, a long-term protocol with OVA aerosols applied every tenth day over 13 weeks did not show an increase in the airway smooth muscle thickness [18]. Thus, it is likely that other factors than merely the exposure time, such as intensity of exposure to antigens, are important aspects. Another possibility is that the composition of complex antigens, such as HDM and CDE, is the reason for the prominent increase in airway smooth muscle thickness. Moreover, several studies of childhood asthma have pointed out that the changes in the airway walls are early events in the innate progression of the disease [22].

Mucosal fibrosis and goblet cell metaplasia and hyperplasia in the airway epithelium are often observed in patients with asthma, and they have been recognized as markers of lung remodelling [23, 24]. In the current study, both subepithelial collagen and mucus-producing cells were increased after exposure to HDM after all three time points, whereas CDE had a significant effect only after 8 and 12 weeks of exposure. In guinea pig OVA models, these features have been shown after 15 days [25] or after 13 weeks of OVA exposure and after 7 weeks of HDM exposure [3]. Thus, this suggests that the heterogeneity in allergen compositions influences both the type and time needed to observe the advancement of structural changes.

Using both Astra blue staining and immunohistochemical detection with tryptase and chymase antibodies, the current study shows that mast cells are present both in the airways and parenchyma of guinea pig lungs in control animals, similar to the baseline condition observed in human lung tissue [16]. The apparent greater amount of mast cells for the Astra blue staining in the control conditions can either be due to the differences in staining techniques or the quantification methods. Immunohistochemistry analysis showed that the main portion of the mast cells was both tryptase- and chymase-positive (MC_TC_), as identified in consecutive sections. This finding differs from lung studies for human healthy individuals where the main part of mast cells are only tryptase-positive (MC_T_) [16, 26, 27]. Thus, although guinea pigs share a similar appearance of mast cells in the lungs, species-specific differences obviously add to the complexity to compare results from different sources. Concerning the granule protease, guinea pigs also show a phenotype more similar to human mast cells than what has been observed in other rodents, including the mouse and rat, where mucosal mast cells only express chymases and no tryptase [8, 9], which also in this aspect indicates that guinea pigs are better animal models than other rodents.

We further investigated the effect of HDM and CDE allergen exposure on mast cell numbers. The HDM- and CDE-stimulated mast cells displayed a strong staining intensity, which was similar to PBS-treated controls, suggesting that secretory granules were intact both in control and treated tissues. This is in line with rapid recycling of mast cell granules and that allergens can repetitively activate mast cells [28, 29]. Using Astra blue staining, it was observed that both allergens caused an increase of mast cells after 8 weeks, which was maintained after 12 weeks, whereas neither of the allergens caused an increase in the number of mast cells after 4 weeks. Therefore, the defining of mast cell phenotypes through immunohistochemistry was performed at 8- and 12-week time points. At these time points, a significant increase of tryptase-positive cells (MC_T_) was found both in the airways and in the parenchyma. Mast cells containing both tryptase and chymase (MC_TC_) showed a minor increase in airways after 8-week exposure. The present increase of mast cells in the airways is in accordance with a higher level of mast cells in the smooth layer of lung tissue in humans with asthma, which as such correlates to an increase of AHR [30, 31]. Furthermore, the shown increase of mast cells in the parenchyma, dominated by MC_T_, mimics the increased infiltration of mast cells in patients with atopic uncontrolled asthma [27]. Although further studies are needed to define in detail the mast cell function in the guinea pig asthma model, our here-described novel model gives a unique opportunity for further studies to better understand the regulation of mast cell accumulation in the lungs upon allergen challenges.

Although most of the inhaled allergens are deposited in the conducting airways, common allergens, like both HDM and cat allergens, may well be transported all the way to the alveolar region [32, 33]. Only a few studies have previously explained the HDM-elicited allergic reactions in guinea pigs [34, 35], and structural changes in large and small airways induced by this allergen have remained unexplored. On the other hand, given that a guinea pig model for cat allergen exposure has not thus far been established, this is also the first evidence that CDE exposure leads to the development of airway remodelling in the guinea pig. Both allergens caused increased inflammation, remodelling, and mast cell increase, but with some significant individual differences. This suggests that the composition of proteases and innate inflammatory activators for HDM and CDE differentially drive airway inflammation. House dust mite and cat allergens are highly immunogenic substances and contain several allergens and molecules that stimulate both innate and adaptive immunity [10, 11]. The mite *Der p 2* and *Der p 7* and cat *Fel d 1* allergens activate epithelial cells through TLR signalling and stimulate alarmin production, thus promoting type 2 inflammation [36, 37]. Particularly, the protease activity caused by HDM leads to impaired barrier function, which influences inflammation and which may be one reason for the different and generally stronger responses for this allergen compared to CDE.

In summary, we have for the first time described the effects of the clinically relevant allergens, HDM and CDE, on the airway structure in the guinea pig model. It is of interest that the investigated features showed the development of allergen-specific patterns over time, indicating that numerous and variable factors may influence inflammation and remodelling. In particular, the guinea pig model gives an opportunity to study both the regulation of mast cells and their increased number in the lungs as well as the role of mast cells in different features of airway inflammation and remodelling to different antigens in asthma.

## 5. Conclusions

In this paper, we show that exposure to HDM and CDE elicits asthma-like histopathology in guinea pigs with infiltration of inflammatory cells, airway remodelling, and accumulation of primarily mucosal mast cells. The results together encourage the use of HDM and CDE allergens for the stimulation of clinically relevant asthma models in guinea pigs.

## Data Availability

The associated data will be available upon reasonable request to the corresponding author.
